# Office Posture Detection Using Ceiling-Mounted Ultra-Wideband Radar and Attention-Based Modality Fusion

**DOI:** 10.3390/s25165164

**Published:** 2025-08-20

**Authors:** Wei Lu, Christopher Bird, Moid Sandhu, David Silvera-Tawil

**Affiliations:** 1 Australian e-Health Research Centre, Commonwealth Scientific and Industrial Research Organisation (CSIRO), Brisbane, QLD 4029, Australia; wei.lu@csiro.au (W.L.);; 2 School of Electrical Engineering and Computer Science, The University of Queensland, Brisbane, QLD 4072, Australia; 3 School of Biomedical Sciences, The University of Queensland, Brisbane, QLD 4072, Australia; 4 School of Computer Science, Queensland University of Technology, Brisbane, QLD 4000, Australia; 5 International Centre for Future Health Systems, University of New South Wales, Sydney, NSW 2052, Australia

**Keywords:** ultra-wideband radar, human activity recognition, signal processing, machine learning, multimodal fusion, office ergonomics

## Abstract

Prolonged sedentary behavior in office environments is a key risk factor for musculoskeletal disorders and metabolic health issues. While workplace stretching interventions can mitigate these risks, effective monitoring solutions are often limited by privacy concerns and constrained sensor placement. This study proposes a ceiling-mounted ultra-wideband (UWB) radar system for privacy-preserving classification of working and stretching postures in office settings. In this study, data were collected from ten participants in five scenarios: four posture classes (seated working, seated stretching, standing working, standing stretching), and empty environment. Distance and Doppler information extracted from the UWB radar signals was transformed into modality-specific images, which were then used as inputs to two classification models: *ConcatFusion*, a baseline model that fuses features by concatenation, and *AttnFusion*, which introduces spatial attention and convolutional feature integration. Both models were evaluated using leave-one-subject-out cross-validation. The *AttnFusion* model outperformed *ConcatFusion*, achieving a testing accuracy of 90.6% and a macro F1-score of 90.5%. These findings demonstrate the effectiveness of a ceiling-mounted UWB radar combined with attention-based modality fusion for unobtrusive office posture monitoring. The approach offers a privacy-preserving solution with potential applications in real-time ergonomic assessment and integration into workplace health and safety programs.

## 1. Introduction

Prolonged sedentary behavior, a defining characteristic of modern office work, has been consistently linked to a range of adverse health outcomes, including musculoskeletal disorders, metabolic dysfunction, and premature mortality. Office workers typically spend approximately 65–75% of their working hours seated, often maintaining prolonged static postures that contribute to discomfort and chronic musculoskeletal pain, particularly in the neck, shoulders, and lower back [1]. In response, incorporating stretching and light activity into the workday has been shown to alleviate musculoskeletal discomfort and improve overall worker well-being. Structured workplace programs that incorporate reminder-based interventions have been particularly effective in promoting compliance with recommended stretching and movement routines. For instance, reminder software has been shown to increase the frequency of stretch breaks, reduce perceived pain, and improve compliance with ergonomic health practices among computer users [2,3]. Moreover, randomized controlled studies and longitudinal workplace implementations have demonstrated that regular stretching sessions, guided by reminder software, structured group breaks, or device-assisted programs, can lead to a decrease in the prevalence of musculoskeletal disorders, particularly in the neck, shoulders, and lower back [4,5].

Recent advancements in sensing technologies have enabled a variety of systems designed to recognize sitting posture and promote office stretching, helping to mitigate risks associated with sedentary work styles. Camera-based solutions have been widely explored for workplace posture and stretch detection. Adolf et al. developed a system that utilizes a single RGB webcam and real-time pose estimation to evaluate stretching performance and provide augmented mirror feedback [6]. Similarly, Paliyawan et al. employed an RGB-D camera system to monitor skeletal posture and identify periods of prolonged sitting. This system classified motion states and offered real-time ergonomic feedback, demonstrating the feasibility of camera-based tracking for sedentary behavior monitoring [7]. In addition to camera-based methods, researchers have also investigated non-visual sensing approaches for posture monitoring in office environments. For example, Tavares et al. and Odesola et al. developed instrumented office chairs equipped with pressure mats or optical fiber arrays capable of detecting pressure distribution patterns and classifying seated postures [8,9]. Zhang et al. proposed a multimodal approach combining pressure and infrared sensors to enhance posture recognition while maintaining user privacy [10]. In a follow-up study, Zhang et al. integrated pressure and spatial temperature data to further improve classification performance [11]. While these methods provide effective posture recognition, they are subject to several limitations. Camera-based systems raise privacy concerns, are susceptible to occlusion in cluttered workspaces, and often require careful sensor placement and lighting conditions. Smart chair systems, while unobtrusive, are restricted to seated postures and cannot effectively capture dynamic stretch-related movements such as arm raises or standing stretches. Furthermore, the multimodal systems, particularly those with sensors mounted vertically on desks or walls, are limited in their ability to capture transitional or full-body stretching due to restricted fields of view and potential occlusion by furniture [9,12].

To address these limitations, we propose a ceiling-mounted ultra-wideband (UWB) radar system for office stretch detection that balances unobtrusiveness, privacy preservation, and robust posture monitoring. UWB radar is a low-power, high-resolution sensing technology that operates by emitting short-duration electromagnetic pulses and analyzing their reflections to detect the motion and position of objects in the environment. While existing UWB radar studies have primarily focused on applications such as sleep posture monitoring, fall detection, and human pose estimation, their findings demonstrate the potential of UWB radar for sensing posture-related activities. For instance, Lai et al. demonstrated that a dual UWB radar configuration can accurately classify sleep postures, showing the technology’s ability to detect subtle body movements from above, even under occlusion and varying environmental conditions [13]. Similarly, Lu et al. employed a ceiling-mounted UWB radar to detect falls in cluttered indoor environments, achieving high classification accuracy through convolutional neural networks trained on distance-time waveform images, suggesting that ceiling-mounted radars can effectively capture complex full-body transitions [14]. Zhou et al. further extended the capabilities of UWB radar by demonstrating micro-Doppler-based human pose estimation, capturing limb-specific kinematic signatures without requiring direct line-of-sight [15]. Collectively, these studies demonstrate the capacity of UWB radar systems to unobtrusively monitor diverse postural behaviors, suggesting their suitability for detecting workplace stretching activities from an overhead perspective. Building on this foundation, our work introduces a ceiling-mounted UWB radar system specifically designed to classify both working and stretching activities in office environments. By leveraging both distance and Doppler signal information captured from an overhead perspective, our system differentiates five workplace scenarios: seated working, seated stretching, standing working, standing stretching, and empty environment.

The main contributions of this study are as follows:

- This study introduces a ceiling-mounted UWB radar configuration tailored for office environments. Unlike prior systems for office posture detection using desk-mounted devices [7,10,11,12] or chair sensors [8,9,10,11,12], this overhead setup offers a wide field of view and reduces potential occlusion, enabling robust monitoring of workspace postures.
- The proposed system is specifically designed to detect working and stretching postures in both seated and standing positions. By capturing these real-world office behaviors, the system enables practical monitoring to support ergonomic interventions.
- The classification model utilizes distance and Doppler information from radar signals and achieved a testing accuracy of 90.6% with leave-one-subject-out cross-validation.

## 2. Materials and Methods

### 2.1. Radar Configurations

**Figure 1:**
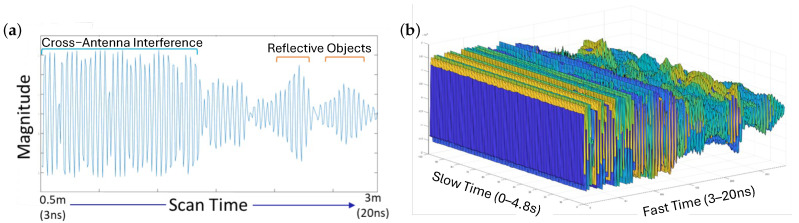
Examples of UWB radar signals: (**a**) a single radar frame illustrating cross-antenna interference and multiple reflected returns from surrounding objects; (**b**) consecutive radar frames collected over a 4.8 s interval. Each frame captures the reflected signal in fast time, and the frames are stacked along the slow time axis to show a longer duration.

The UWB radar used in this study is a compact monostatic UWB radar module (P440, TDSR LLC., Petersburg, VA, USA) that includes a programmable radar unit and two planar elliptical dipole antennas. Detailed specifications for the radar and antennas are provided in Table 1 and Table 2. Operating as a monostatic radar, a short-duration, low-power electromagnetic pulse is emitted by the transmitting antenna, and the receiving antenna captures the reflected signals from objects in the environment. Operating in a monostatic setup, the radar system emits short-duration, low-power electromagnetic pulses via the transmitting antenna. The receiving antenna, co-located with the transmitter, captures the reflected signals from objects within the environment. The time-domain response of these reflections, with amplitude recorded as a function of propagation delay, forms a single radar frame. Each frame may include components from cross-antenna interference as well as reflections from targets located at varying distances. A typical signal frame is shown in Figure 1a. The temporal axis of a single frame, referred to as fast time, is measured in nanoseconds and corresponds to the propagation delay of the transmitted pulse, which directly relates to the distance of objects from the radar. The radar system captures one frame every 12 ms, corresponding to a frame rate of approximately 83.3 Hz. When multiple frames are acquired sequentially and aligned over time, they form a two-dimensional matrix in which the second axis, referred to as slow time, captures changes over longer durations, typically on the order of seconds. Figure 1b illustrates an example of consecutive frames acquired over a 4.8 s interval in slow time, with each frame capturing distance-related information in fast time.

**Figure 2:**
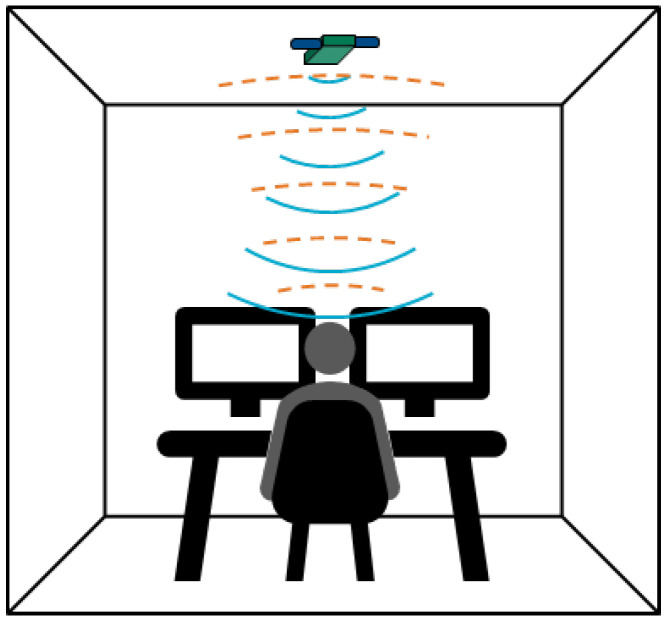
Setup of the ceiling-mounted UWB radar positioned above a standard office workstation. The radar is oriented downward to capture movement and posture within the workspace, accommodating both seated and standing desk configurations.

For posture monitoring, the radar was mounted on the ceiling directly above the office worker’s desk, with its antennas oriented downward to capture movement and posture within the workspace, as shown in Figure 2. The room measured 5.0m×5.0m×2.6m (length, width, height), with the desk positioned at the center of the room. The desk height was set to 0.75m for the sitting configuration and 1.0m for the standing configuration. The office chair had a seat height of 0.5m. It should be noted that the scan duration for a single frame was configured to allow a maximum path distance of 6 m (i.e., 3 m in one direction), exceeding the distance from the radar to the participant and desk setup. A laptop was connected to two monitors placed on the desk, along with a keyboard and a mouse, to simulate a typical office workstation. The radar was connected to a computer running a custom software tool designed for data acquisition.

### 2.2. Data Collection

Posture detection using UWB radar in an office environment was formulated as a five-class classification task, using the following categories: seated working, seated stretching, standing working, standing stretching, and empty environment (i.e., when no person is present in the monitored area). These classes were selected to capture typical workplace behaviors relevant to sedentary risk assessment and ergonomic intervention [1,16].

To collect training and evaluation data for the posture classification model, a data collection trial was conducted with approval (2023_007_R) from the Health and Medical Human Research Ethics Committee of Commonwealth Scientific and Industrial Research Organisation (CSIRO). Ten adult participants (5 males and 5 females; average age 26.5±7.1 years) were recruited to complete a protocol simulating office work and stretching activities. Eligibility criteria included being at least 18 years of age, able to read and speak English, and physically capable of performing light stretching and standing tasks. Individuals with pre-existing injuries or mobility restrictions were excluded from participation.

**Figure 3:**
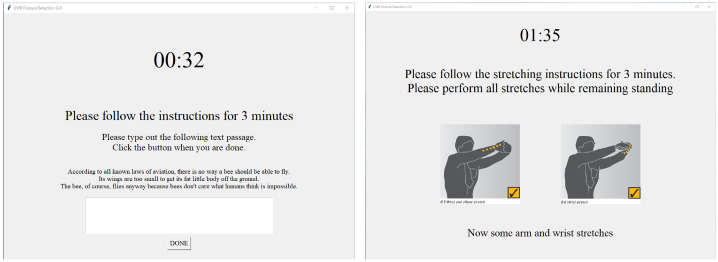
GUI used during data collection. The interface guided participants through a sequence of four posture tasks: seated working, seated stretching, standing working, and standing stretching, with each task lasting three minutes.

**Figure 4:**
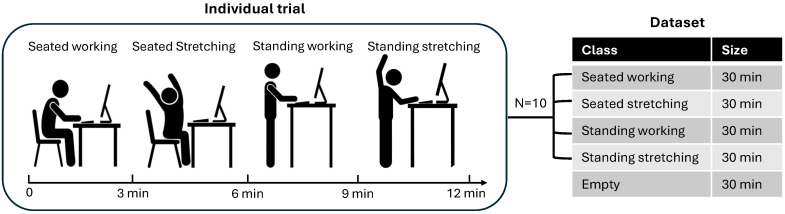
Overview of the data collection process. Ten participants (N = 10) were guided by a custom GUI to perform each posture class for 3 min. Additionally, 30 min of data were recorded under the “empty” condition, representing an unoccupied workspace.

**Figure A1:**
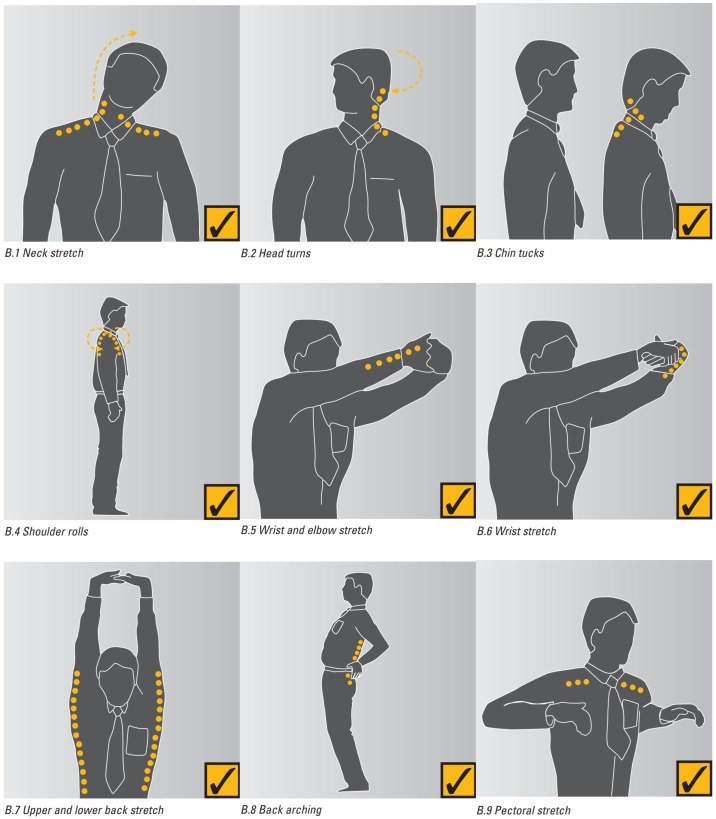
Stretching postures included in the study [17]. Shoulder rolls and back arching, shown as standing postures in the figure, were also performed by participants while seated.

During each trial, the radar system was connected to a computer for data acquisition. A custom graphical user interface (GUI) guided participants through four posture tasks, each lasting 3 min: seated working, seated stretching, standing working, and standing stretching (Figure 3). The working tasks involved typical computer-based interactions such as typing and mouse clicking. The stretching routines were adapted from WorkSafe Victoria’s ergonomic exercise guidelines, which recommend short, simple movements that can be performed without special equipment in typical office settings (e.g., shoulder rolls, pectoral stretch, and head turns) [17]. These guidelines are widely used in workplace health programs in Australia and emphasize stretches targeting the neck, shoulder, and arm areas most affected by prolonged seated or standing computer work. Illustrations of the stretching postures are provided in the Appendix A (Figure A1). Following the seated tasks, the desk was reconfigured to a standing desk to facilitate the standing activities. In addition to the four participant tasks, separate recordings were made under the “empty” condition to represent unoccupied workspace scenarios. An overview of the data collection protocol is summarized in Table 3. In total, the dataset comprised 120 min of posture data, including 12 min per participant across four postures, and 30 min of radar data under empty condition (Figure 4).

### 2.3. Signal Processing

In order to characterize occupancy states and human postures, the collected radar data were processed to generate distance and Doppler images. The distance image captures the changes in spatial reflections over time, providing information about the relative position and movement of objects in the monitored area. The Doppler image captures velocity-related information by representing frequency shifts over time, reflecting the motion dynamics of observed objects.

#### 2.3.1. Distance Image

As the first step in distance image processing, the original radar frames were augmented by sub-sampling to produce four sub-sequences of the data. Specifically, the sub-sampling was performed with a stride of four, starting from different initial frames. Let $X=\{x_{0},x_{1},x_{2},\ldots ,x_{N-1}\}$ denote the original radar frames collected at a frame period of 12 ms. The four sub-sequences were generated by:

(1) $$X^{\left(o\right)}=\left\{x_{o+4k}|k=0,1,2,\ldots \right\},\;o\in \left\{0,1,2,3\right\}$$

Each $X^{\left(o\right)}$ represents a temporally shifted version of the data with an effective frame period of 48 ms. This processing generates four sub-sequences from the same recording window. For example, the 12 min of posture data recorded per participant were sub-sampled into four temporally shifted 12 min sequences, effectively augmenting the data by four times. A distance image was constructed using 4.8 s segments from the sub-sequence, corresponding to 100 frames per image. These segments were extracted using a sliding window with 75% overlap between consecutive windows. To construct a distance image from each 4.8 s segment, every frame within the segment was first band-pass filtered along the fast time axis using a predefined infinite impulse response (IIR) filter, attenuating low-frequency background signals and high-frequency noise. The filtered signal was then processed along the slow time axis using a high-pass finite impulse response (FIR) filter, isolating areas of dynamic activity (e.g., motion due to posture changes). The resulting signal comprises 96 frames, as the first few frames were removed to eliminate edge effects from the FIR filtering. To enhance the signal’s magnitude and emphasize motion-related features, an absolute value transformation was applied, followed by a low-pass IIR filter to extract the motion-related signal envelope over time. Finally, residual low-level noise was attenuated using a non-linear amplitude squashing function resembling a sigmoid function:

(2) $$x_{0}=\frac{x}{1+1.05^{-x+30}}$$

**Figure 5:**
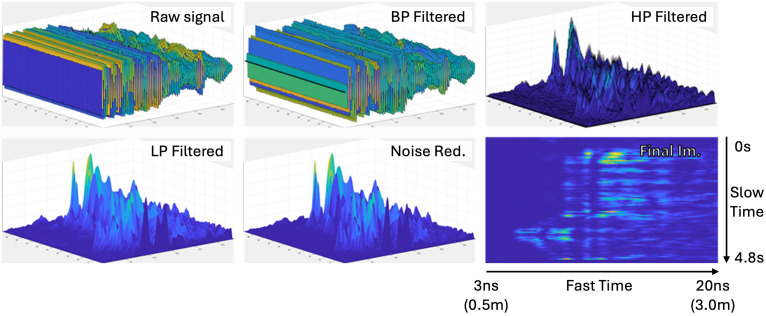
Step-by-step illustration of the distance image generation.

This series of operations produced distance images that represent changes in range intensity over time, capturing body movements relative to the radar. Each image has a final resolution of 96×288, corresponding to the number of retained slow-time frames and fast-time bins after filtering. The complete distance image generation pipeline is shown in Figure 5. In total, 26,000 distance images were extracted from the original radar data, comprising 5200 images per class across the four postures and the empty scenario.

#### 2.3.2. Doppler Image

**Figure 6:**
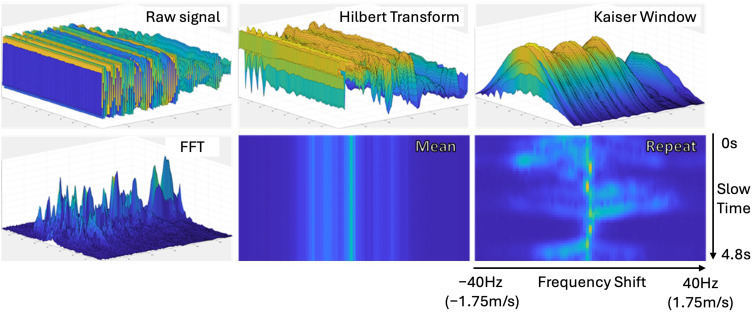
Step-by-step illustration of the Doppler image generation.

Doppler images were generated by first applying a short-time Fourier transform (STFT) to the full duration of each original radar recording, for example, the 12 min posture data from each participant. The STFT was computed using a 996 ms window, corresponding to 83 frames sampled at a 12 ms frame period. Within the STFT window, each frame was processed with a Hilbert transform along the fast time axis, and a Kaiser window (β = 6) was applied along the slow time axis. The STFT was then performed along the slow time axis, and the mean across the fast time axis was used to compute each row of the Doppler image. To improve temporal resolution, an overlap with a 9-frame increment (approximately 90% overlap) was applied between consecutive windows, effectively sliding the STFT window across the entire recording. The resulting Doppler representation shows the relative velocities of objects moving toward or away from the radar over the full recording duration. Finally, Doppler images were extracted as 4.8 s segments from the full Doppler representation using time windows matching those used for distance image construction. The resulting Doppler images have a resolution of 50×80, representing the number of time frames and Doppler frequency bins extracted from the STFT. The Doppler image generation pipeline is illustrated Figure 6.

#### 2.3.3. Normalization

Both the distance and Doppler images were normalized to the range 0–255 and saved as 8-bit single-channel images. These normalized images were then used as input to the classification models to distinguish between different postures and the empty environment.

### 2.4. Classification Models

The classification task aims to predict the class label based on dual-modality radar inputs: a distance image and a Doppler image. Each sample is represented as $X=(X_{d},X_{v})$, where $X_{d}\in R^{1\times 96\times 288}$ is the single-channel distance image and $X_{v}\in R^{1\times 50\times 80}$ is the corresponding Doppler image. The associated label y∈Y denotes one of five classes: seated working, seated stretching, standing working, standing stretching, or empty environment. The training objective is to learn a function $f:X=(X_{d},X_{v})\to y$ that maps each sample X to its corresponding class label *y*. Two deep learning models were developed for this classification task. Both models adopt a dual-stream convolutional neural network (CNN) architecture, in which modality-specific features are extracted from Doppler and distance representations using separate CNN blocks. The first model, *ConcatFusion*, implements a feature-level fusion approach in which modality-specific representations are concatenated prior to classification. The second model, *AttnFusion*, enhances this design by introducing spatial attention modules to emphasize informative regions in each modality and a deeper fusion block that integrates the refined features before classification. These models were implemented using Python 3.10.7 and PyTorch Lightning 2.5.1. Model performance was evaluated using leave-one-subject-out cross-validation (LOSO-CV), with accuracy, F1-score, precision, and recall as performance metrics.

#### 2.4.1. *ConcatFusion*

**Figure 7:**
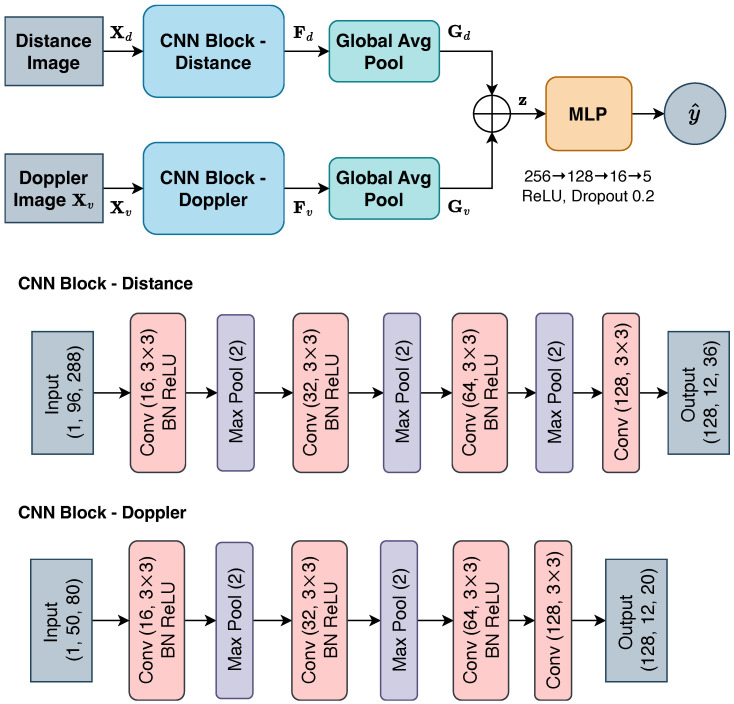
Architecture of the *ConcatFusion* model.

The *ConcatFusion* model adopts a dual-stream CNN architecture that processes Doppler and distance images independently before fusing the learned features for classification. The architecture of the model is shown in Figure 7. The input images $X_{d},X_{v}$ are passed through modality-specific CNN blocks:

(3) $$F_{d}=f_{d}\left(X_{d}\right),\;F_{v}=f_{v}\left(X_{v}\right)$$

where $f_{d}\left(\cdot \right)$ and $f_{v}\left(\cdot \right)$ are modality-specific CNN blocks, producing intermediate feature maps, $F_{d}\in R^{128\times 12\times 36}$ and $F_{v}\in R^{128\times 12\times 20}$. The Doppler CNN block uses a shallower architecture than the distance CNN block to account for the smaller input size of Doppler images. Each intermediate feature map is then passed through a global average pooling layer to generate global representations $G_{d},G_{v}\in R^{128\times 1\times 1}$. These are flattened to form the modality-specific embeddings $z_{d},z_{v}\in R^{128}$, which are then concatenated to produce a joint feature vector:

(4) $$z=\left[z_{d};z_{v}\right]\in R^{256}$$

The fused vector z is passed through a multilayer perceptron (MLP) classifier to produce the final classification result over the five predefined classes.

#### 2.4.2. *AttnFusion*

**Figure 8:**
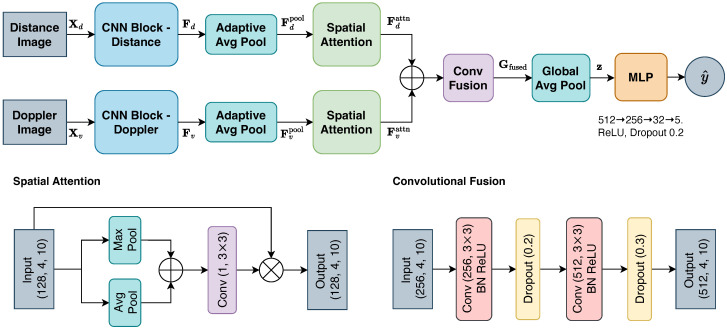
Architecture of the *AttnFusion*.

The *AttnFusion* model extends the *ConcatFusion* architecture by incorporating spatial attention modules and a convolutional fusion block to enhance multimodal feature integration. The architecture of the model is shown in Figure 8. The distance and Doppler images are first passed through the same modality-specific CNN blocks as those used in the *ConcatFusion* model (Equation (3)). The outputs of the CNN blocks, $F_{d},F_{v}$, are passed through an adaptive average pooling layer to produce fixed-size feature maps $F_{d}^{\mathrm{pool}}$, $F_{v}^{\mathrm{pool}}\in R^{128\times 4\times 10}$. These pooled maps are then refined by a spatial attention module that creates a spatial attention mask that highlights informative regions by weighting spatial locations based on channel-wise statistics [18]. Specifically, the module first applies average and max pooling across the channel dimension, concatenates the results, and passes them through a shared convolutional layer with a 3×3 kernel and sigmoid activation. This convolutional layer has 2 input channels, corresponding to the pooled average and max features, and produces a single-channel attention mask $M_{s}\left(F\right)\in R^{1\times 4\times 10}$:

(5) $$\begin{array}{cc} M_{s}\left(F\right) & =\sigma (f^{3\times 3}\left([\mathrm{AvgPool}(F);\mathrm{MaxPool}(F)]\right))\\ \; & =\sigma \left(f^{3\times 3}\left([F_{s}^{\mathrm{avg}};F_{s}^{\max}]\right)\right),\;F\in \left\{F_{d}^{\mathrm{pool}},F_{v}^{\mathrm{pool}}\right\} \end{array}$$

Then, $F_{d}^{\mathrm{pool}}$ and $F_{v}^{\mathrm{pool}}$ are multiplied element-wise with their corresponding attention mask across all channels to produce the attention-refined output, $F_{d}^{\mathrm{attn}}$, $F_{v}^{\mathrm{attn}}\in R^{128\times 4\times 10}$, respectively. The attention-refined distance and Doppler features are concatenated along the channel dimension to form a fused representation:

(6) $$F_{\mathrm{fused}}=\left[F_{d}^{\mathrm{attn}};F_{v}^{\mathrm{attn}}\right]\in R^{256\times 4\times 10}$$

This fused representation is processed by a convolutional fusion block, followed by global average pooling to generate a global presentation $G_{\mathrm{fused}}\in R^{512\times 1\times 1}$. The output is then flattened to form a feature vector $z\in R^{512}$, which is passed through an MLP to produce the final classification result over the five predefined classes.

#### 2.4.3. Training and Evaluation

Model performance was evaluated using LOSO-CV across 10 folds. In each fold, data from 10 participants were split into three groups: 8 for training, 1 for validation, and 1 for testing. This setup ensured that each participant was included once as the validation subject and once as the test subject, allowing for balanced evaluation across individuals. During each fold, the model was trained on the training subset, while the validation set was used to monitor convergence and apply early stopping to prevent overfitting. The model that achieved the highest validation accuracy was then evaluated on the held-out test subset. This process was repeated 10 times, producing 10 sets of performance metrics for each fold. The training hyperparameters are listed in the Appendix A (Table A1).

Performance metrics included accuracy, macro precision, macro recall, and macro F1-score, computed on the test subset of each fold. Let $TP_{k}$, $FP_{k}$, and $FN_{k}$ denote the number of true positives, false positives, and false negatives for class *k*, respectively. Let *K* be the number of classes and *N* the total number of test samples. The accuracy is defined as:

(7) $$\mathrm{Accuracy}=\frac{1}{N}\sum_{k=1}^{K}TP_{k}$$

Macro precision, recall, and F1-score were computed by first evaluating each class independently and then averaging over the K=5 classes. For class *k*, precision and recall are defined as:

(8) $${\mathrm{Precision}}_{k}=\frac{TP_{k}}{TP_{k}+FP_{k}},\;{\mathrm{Recall}}_{k}=\frac{TP_{k}}{TP_{k}+FN_{k}}$$

Macro precision, recall, and F1-score are then computed as:

(9) $$\mathrm{Macro}\;\mathrm{Precision}=\frac{1}{K}\sum_{k=1}^{K}{\mathrm{Precision}}_{k},\;\mathrm{Macro}\;\mathrm{Recall}=\frac{1}{K}\sum_{k=1}^{K}{\mathrm{Recall}}_{k}$$

(10) $$\mathrm{Macro}\;F1=\frac{1}{K}\sum_{k=1}^{K}\frac{2\cdot {\mathrm{Precision}}_{k}\cdot {\mathrm{Recall}}_{k}}{{\mathrm{Precision}}_{k}+{\mathrm{Recall}}_{k}}$$

To further assess the discriminative performance of the models for each class, aggregate receiver operating characteristic (ROC) curves were generated by combining all test samples from the 10 LOSO-CV folds. For each class, the ROC curve and corresponding area under the curve (AUC) were computed based on the aggregated test results, providing a summary of class-wise discriminative ability across all participants.

## 3. Results

This section presents the dataset details and overall model performance, including accuracy, F1-score, precision, and recall. Further analyses include per-class metrics, confusion matrices, ROC curves, and per-participant performance.

### 3.1. Dataset

A total of 26,000 samples were obtained from preprocessing the trial data, where each sample is represented as a pair of distance and Doppler images $X=(X_{d},X_{v})$, along with a corresponding class label *y*. The dataset includes five categories: four postures (seated working, seated stretching, standing working, and standing stretching) and one empty condition, with 5200 samples per class. Each of the 10 participants contributed 520 samples per class, resulting in 2600 samples per participant. In the LOSO-CV setup, each fold uses 20,800 samples from 8 participants for training, 2600 samples from 1 participant for validation, and 2600 samples from 1 participant for testing.

### 3.2. Overall Performance

Overall performance was evaluated by averaging the test results across 10 test folds, with each fold corresponding to one participant held out for testing (Table 4). The *AttnFusion* model outperformed *ConcatFusion* across all metrics, achieving a higher accuracy (90.6±4.2%) and F1-score (90.5±4.3%). In comparison, *ConcatFusion* achieved an accuracy of 85.5±5.8% and an F1-score of 85.4±6.0%, with a higher standard deviation across participants.

### 3.3. Per-Class Performance

**Figure 9:**
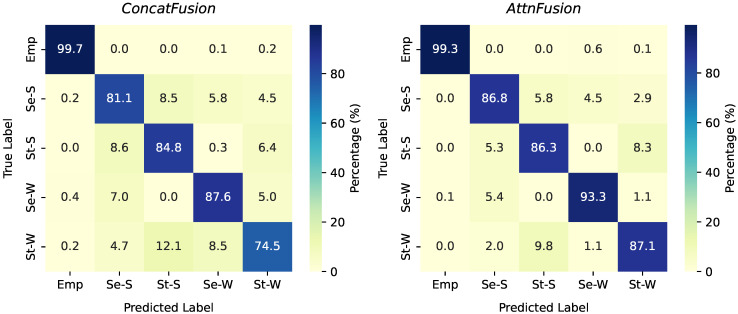
Cumulative confusion matrices for the *ConcatFusion* (**left**) and *AttnFusion* (**right**) models. The class labels are shown as: Emp (empty), Se-S (seated stretching), St-S (standing stretching), Se-W (seated working), and St-W (standing working).

Confusion matrices were computed by aggregating the classification results across all test folds in LOSO-CV, showing the true labels versus predicted labels for each class (Figure 9). For the four posture classes, the *AttnFusion* model achieved higher diagonal values, indicating improved sensitivity for each class compared to the *ConcatFusion* model. Both models showed high sensitivity for the empty classes, with over 99% of empty samples classified correctly. When classifying seated stretching, *AttnFusion* achieved an accuracy of 86.8%, compared to 81.1% with *ConcatFusion*, reducing misclassification as standing stretching and working classes. A similar trend was observed for standing stretching, with *AttnFusion* achieving 86.3% correct classification versus 84.8% for *ConcatFusion*. Both models showed some confusion between stretching and working postures within the same desk configuration (seated or standing); however, this was less evident in the *AttnFusion* model. For seated working, *AttnFusion* classified 93.3% of instances correctly, compared to 87.6% for *ConcatFusion*, and for standing working, *AttnFusion* reached 87.1%, a significant improvement over *ConcatFusion*’s 74.5%. Off-diagonal values indicate that errors most often involved confusion between stretching and working postures in the same physical position, rather than between seated and standing. Overall, *AttnFusion* consistently improved per-class classification performance, particularly for working classes.

**Figure 10:**
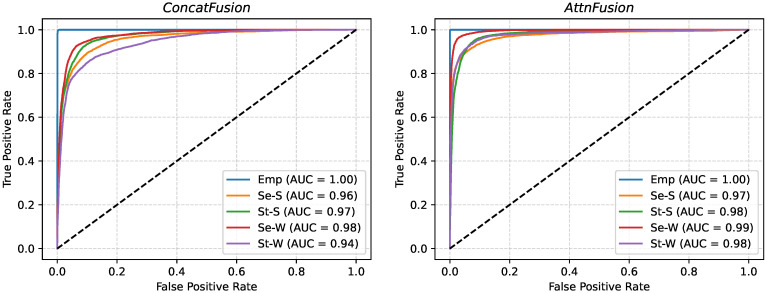
Aggregate ROC curves by class for the *ConcatFusion* (**left**) and *AttnFusion* (**right**) models. The class labels are shown as: Emp (empty), Se-S (seated stretching), St-S (standing stretching), Se-W (seated working), and St-W (standing working).

ROC curves were computed for each class by combining all test samples across the 10 LOSO-CV folds (Figure 10). Both models achieved high AUC values for the empty class (AUC = 1.00). The *AttnFusion* model consistently outperformed *ConcatFusion* across the four posture classes, most notably for standing working (AUC = 0.98 vs. 0.94). Collectively, these results demonstrate robust class-wise discriminative ability and the benefits of modality-specific spatial attention and convolutional modality fusion in the *AttnFusion* model.

### 3.4. Per-Participant Performance

**Figure 11:**
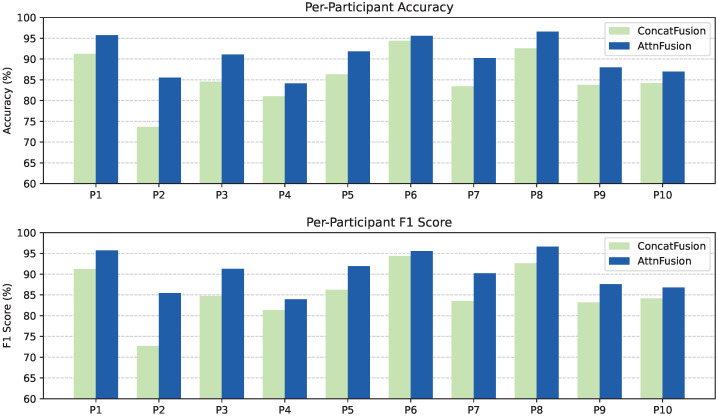
Per-participant accuracy (**top**) and F1-score (**bottom**) for the *ConcatFusion* and *AttnFusion* models. Each cluster of bars corresponds to one participant (P1–P10).

For each participant, accuracy and F1-score for both the *ConcatFusion* and *AttnFusion* models are reported in Figure 11. The *AttnFusion* model outperformed *ConcatFusion* for most participants. Notably, several participants (e.g., P2 and P3) showed larger gains with the *AttnFusion* model. While some inter-participant variability was observed, the overall trend indicates stable performance across individuals and consistent benefits from the *AttnFusion* model.

## 4. Discussion

This study demonstrates that a ceiling-mounted UWB radar system can differentiate both working and stretching postures in an office environment by combining distance and Doppler information. The *AttnFusion* model demonstrated better overall performance, achieving a testing accuracy of 90.6% ± 4.2% and macro F1-score of 90.5% ± 4.3%, compared to 85.5% ± 5.8% and 85.4% ± 6.0% for *ConcatFusion*. The relatively lower standard deviation observed with *AttnFusion* also indicates greater consistency across different participants. At the class level, *AttnFusion* improved the classification of challenging postures, with seated stretching increasing from 81.1% to 86.8% and standing working from 74.8% to 87.1%, while maintaining high accuracy for other classes. When examined per participant, *AttnFusion* delivered more stable performance, with reduced variability in all performance metrics across all participants, highlighting its robustness to inter-individual differences. These results suggest that combining spatial attention with convolutional feature integration in a dual-modality framework can substantially improve the reliability of UWB radar-based posture recognition in office environments.

Our findings align with and extend recent radar-based human posture recognition research. Liu et al. achieved high accuracy in classifying five seated postures using FMCW radar, reporting an average accuracy above 98% [19]. Similarly, Zhao et al. proposed a deep learning pipeline for human motion and posture recognition using mmWave imaging radar, achieving robust angle-insensitive recognition by fusing point cloud and spectrogram representations [20]. Their system achieved an overall accuracy of 87.1% for six activities, including sitting down, standing up from sitting, bending over, standing up from bending, sitting still, and standing still. Zhang et al. evaluated several machine learning classifiers for posture identification using point clouds from FMCW radar [21]. Their best-performing MLP classifier achieved 94% accuracy in differentiating six postures, including sitting, lying, and four different standing postures. Additionally, some previous studies considered standing and sitting as two broad classes, without further dividing them into specific postural subclasses [22,23,24]. In comparison to the prior work, this study differs in several key aspects. First, in terms of activity recognition, our system aims to distinguish working and stretching postures in seated and standing conditions, but previous studies have often focused on a subset of postures, such as sitting postures or daily activities. Second, we employed a LOSO-CV protocol to assess performance and generalizability across different participants, while most previous studies on radar-based posture recognition relied on random splits of data from all participants or used data from a single individual, which may overestimate model generalization [19,21,22,23,24]. Finally, our use of a ceiling-mounted UWB radar offers unobtrusive monitoring with minimal occlusion, in contrast to previous studies employing side-mounted radars on desks or tripods, thereby enhancing practicality for office deployment [19,20,21,22,23]. Table 5 summarizes previous radar-based human posture recognition studies, comparing sensor location, number of participants, covered postures, validation methods, and reported accuracies.

The proposed radar-based approach offers several advantages over conventional camera-based and wearable office posture detection systems. Compared to camera-based systems, it enhances privacy by avoiding the capture of identifiable visual information and maintains consistent performance under varying lighting conditions, including low-light or dark environments [25]. Compared to wearable devices, the radar-based system requires no user compliance or physical attachment, allowing continuous monitoring without interfering with daily activities. In addition, UWB radar offers low power consumption, supporting energy-efficient operation suitable for long-term deployments. It is inherently resistant to narrowband interference, enabling reliable performance in environments with other wireless systems, and can sustain long-term continuous monitoring with minimal maintenance requirements [26].

Future research will build on the findings of this study while addressing its limitations. First, variations in body size and shape can affect the magnitude and distribution of reflected signals, while differences in movement patterns across age groups and genders may influence Doppler patterns. As the current dataset comprised ten participants with a relatively narrow age range, the results may not fully capture the variability present in broader populations. Future research should extend validation to more diverse participant groups, additional office environments, and a wider range of postures and stretching activities to improve the generalizability of the proposed system. Second, while the results demonstrated the feasibility of the proposed system, the signal processing pipeline and classification models were designed empirically. Both can be further optimized through tuning of signal processing parameters (e.g., data segmentation and filter settings), architecture refinement, and model hyperparameter optimization. A more comprehensive optimization process leveraging a larger dataset, incorporating explainable AI methods, and enabling lightweight deployment on edge devices could improve system transparency, performance, and overall utility. Third, the proposed system is designed to cover a single-person workspace, which does not address the presence of multiple individuals. Future work will explore the integration of additional sensing modules or dedicated radar signal processing pipelines capable of distinguishing between single- and multi-person occupancy in shared workspaces. Finally, real-world deployments are needed to assess the impact of environmental factors such as workspace clutter, reflective surfaces, and variations in ceiling height on system performance.

## 5. Conclusions

In this work, we propose a privacy-preserving and unobtrusive system for monitoring ergonomic behaviors in office environments using ceiling-mounted UWB radar. By integrating both distance and Doppler information extracted from UWB radar signals, our approach enables accurate detection of static and dynamic postures while minimizing the privacy concerns and workplace disruptions. This work provides a radar-based solution for scalable workplace interventions aimed at reducing musculoskeletal risks, prompting micro-breaks, and supporting healthier work routines. Further research should explore multi-person scenarios, extended environments, and real-world deployment to improve generalizability and impact.

## Figures and Tables

**Table 1:** Radar specifications.

Parameter	Value
Operating band	3.1–4.8 GHz
Pulse repetition rate	10 MHz
Transmission power	~50 µW
Transfer switch isolation	~20 dB
Receive noise fatigue	~4.8 Hz

**Table 2:** Antenna specifications.

Parameter	Value
Polarization	Vertical
VSWR	~1.75:1
S11	~12 dB
Gain	~3 dBi
Phase response	Linear

**Table 3:** Protocol for the data collection trial.

Class	Description
Seated working	Typing, mouse clicking, and reading while seated
Seated stretching	Stretching exercises while seated
Standing working	Typing, mouse clicking, and reading while standing
Standing stretching	Stretching exercises while standing
Empty	No person present in the workspace

**Table 4:** Overall test performance of the proposed models. Values represent the mean ± standard deviation across 10 test folds.

Model	Accuracy (%)	Precision (%)	Recall (%)	F1-Score (%)
*ConcatFusion*	85.5±5.8	87.0±4.8	85.5±5.8	85.4±6.0
*AttnFusion*	90.6±4.2	91.4±3.7	90.6±4.2	90.5±4.3

**Table 5:** Comparison of previous studies on radar-based human posture recognition.

Study	Sensor Location	Number of Participants	Sitting and Standing ^1^	Working and Stretching ^1^	Subject-Wise Validation	Accuracy ^2^
[19]	Side	5	✘	✘	✘	98.7%
[20]	Side	8	✔	✘	✔	97.9%
[21]	Side	1	✔	✘	✘	94.0%
[22]	Side	5	✔	✘	✘	84.9%
[23]	Side	9	✔	✘	✘	97.1%
[24]	Overhead	3	✔	✘	✘	98.9%
This study	Overhead	10	✔	✔	✔	90.6%

^1^ Types of postures included in the study. ^2^ Accuracy of the best-performing model reported in each study; the calculation of accuracy varies according to the validation method employed.

## Data Availability

Data are unavailable due to privacy or ethical restrictions.

## Abbreviations

The following abbreviations are used in this manuscript:

UWB	Ultra-wideband
CSIRO	Commonwealth Scientific and Industrial Research Organisation
GUI	Graphical user interface
STFT	Short-time Fourier transform
CNN	Convolutional neural network
LOSO-CV	Leave-one-subject-out cross-validation
MLP	Multilayer perceptron
ROC	Receiver operating characteristic
AUC	Area under the curve
Emp	Empty
Se-S	Seated stretching
St-S	Standing stretching
Se-W	Seated working
St-W	Standing working

## Appendix A

**Table A1:** Model training hyperparameters.

Hyperparameter	Value
Optimizer	AdamW
Weight decay	$1\times 10^{-4}$
Batch size	64
Max epochs	200
Loss function	Cross Entropy
Learning rate scheduler	ReduceOnPlateau
Scheduler monitor	Validation loss
Scheduler factor	0.5
Scheduler patience	20
Early stopping monitor	Validation accuracy
Early stopping patience	20
